# Syphilis in pregnancy

**DOI:** 10.12669/pjms.311.5932

**Published:** 2015

**Authors:** Asrul Abdul Wahab, Umi Kalsom Ali, Marlyn Mohammad, Ezura Madiana Md. Monoto, M.M. Rahman

**Affiliations:** 1 Asrul Abdul Wahab, Department of Medical Microbiology & Immunology, Faculty of Medicine, Universiti Kebangsaan Malaysia, Cheras 56000 Kuala Lumpur, Malaysia.; 2 Umi Kalsom Ali, Department of Medical Microbiology & Immunology, Faculty of Medicine, Universiti Kebangsaan Malaysia, Cheras 56000 Kuala Lumpur, Malaysia.; 3 Marlyn Mohammad, Department of Medical Microbiology & Immunology, Faculty of Medicine, Universiti Kebangsaan Malaysia, Cheras 56000 Kuala Lumpur, Malaysia.; 4 Ezura Madiana Md. Monoto, Department of Medical Microbiology & Immunology, Faculty of Medicine, Universiti Kebangsaan Malaysia, Cheras 56000 Kuala Lumpur, Malaysia.; 5 M.M. Rahman, Department of Medical Microbiology & Immunology, Faculty of Medicine, Universiti Kebangsaan Malaysia, Cheras 56000 Kuala Lumpur, Malaysia.

**Keywords:** Congenital syphilis, Pregnancy, Rapid Plasma Reagin, Syphilis IgG

## Abstract

Syphilis in pregnancy remains an important medical condition due to its consequences. We present two cases of young pregnant women who were diagnosed syphilis during their antenatal visit. The first case was a 29-year-old Malay lady diagnosed with syphilis during the first trimester of pregnancy, while the second case was a 21-year-old Chinese lady diagnosed with syphilis during the third trimester of pregnancy. The diagnosis and management of the syphilis in pregnancy are discussed.

## INTRODUCTION

Syphilis is caused by the spirochete *Treponema pallidum *subspecies *pallidum*, which is of particular concern during pregnancy because of the risk of trans-placental infection to the fetus. Stillbirths and early childhood mortality due to syphilis are continually being reported each year. World Health Organization (WHO) estimated that up to 1.5 million cases of syphilis in pregnancy occurs each year.^1^ Timely diagnosis and proper management of the infection in the pregnant woman are important in order to prevent adverse outcome.

**CASE 1: **Mrs. ZNA, a 29-year-old Malay housewife, Gravida 4 Para 2+1, came for antenatal booking at the primary care clinic, complaining of polyuria, polydipsia and lethargy for the past one week. Dating ultrasound revealed 11 weeks fetus. She was diagnosed with gestational diabetes mellitus (GDM) with fasting blood glucose of 11.0 mmol/L and subsequently referred here for further management. She also complained of itchiness at the genital area associated with whitish vaginal discharge whereby the high vaginal swab specimen for microbiology culture revealed presence of candida infection. She was subsequently treated appropriately. 

Routine blood investigations including hepatitis B, human immunodeficiency virus (HIV) and syphilis serology tests were done. The serology tests for hepatitis B and HIV were negative. However, the rapid plasma reagin (RPR) was reactive at 1:16 titration. The diagnosis of syphilis was confirmed by a positive Syphilis IgG result. 

On further history, she admitted to the treatment of syphilis during her previous pregnancy in 2010 at another hospital. She was given three doses of intramuscular penicillin. Previous syphilis record showed the RPR titre was 1:8 but no subsequent follow-up.

The diagnosis of syphilis re-infection was made and she was treated with 2.4 million units of penicillin weekly for three doses. Her other medical problems were managed accordingly. She was discharged from the ward once the blood sugar level was optimized and continued her follow up in the clinic. Her husband was counselled for syphilis screening but refused.

Consequently, she completed the treatment for syphilis. The second and third trimester ultrasounds revealed no abnormalities. Repeated RPR at 33 weeks of gestation was non-reactive.

She delivered a baby boy at 38 weeks of gestation through LSCS with birth weight of 4.0 kg. No clinical signs of congenital syphilis noted. Rapid Plasma Reagin (RPR) result for the baby was non-reactive. She was discharged after three days in the ward. Post-natal follow up was scheduled for them but she requested to be seen in another hospital at her hometown.

**CASE 2:**Mrs. TPS is a 21-year-old Chinese housewife, Gravida 1 Para 0, at 31 weeks gestation was admitted to the ward for premature contraction. She gave a 3-days history of reduced fetal movement.

Antenatally, she attended antenatal check up in another hospital. She was mildly anaemic with haemoglobin of 10.8 g/dL and was treated with oral haematinics. Otherwise it was uneventful. She recently moved to Kuala Lumpur, hence had never attended antenatal follow up in this hospital. Both her and her husband, a 21-year old chef denied any high-risk behavior in the past.

On arrival, she was already in advanced labor and delivered a macerated stillbirth baby boy, weighing 1.48 kg. Grossly it looked normal with no facial dysmorphism.Blood investigation taken during admission noted that her RPR was reactive at 1:64 titrations, with positive syphilis IgG antibody. She was explained about syphilis and pregnancy and offered treatment but she requested to follow-up in another hospital. Her husband was also counseled but did not agreed for blood testing.

## DISCUSSION

Syphilis is one of the sexually transmitted infections. World Health Organization (WHO) estimates nearly 1.5 millions of pregnant women are infected with probable active syphilis each year and approximately, half of the untreated pregnant women suffer adverse outcome during pregnancy.^1^ Antenatal screening for syphilis provides a good opportunity to detect the disease early. Those who attended antenatal care but were not offered syphilis testing have been shown to have adverse outcome of the disease.^2^ In Malaysia, antenatal screening test for syphilis by non-treponemal serology test is recommended during the first visit and subsequently at 28 week of gestation.^3^

Syphilis can be divided into several stages: primary, secondary, latent and tertiary syphilis. Clinical manifestations of syphilis are not apparently altered by pregnancy.^4^ Vertical transmission can occur at any time and stage of syphilis. Risk of transmission correlates with the extent of spirochetes presence in the blood circulation, thus primary and secondary syphilis carry a higher risk of transmission than latent and tertiary syphilis.^5^ The lesions of primary syphilis occur about three weeks after sexual contact and they are often unrecognized in women because they can be asymptomatic.^5^ Based on clinical history obtained, both of our cases were probably at the early stage of syphilis (primary, secondary or early latent).

 Congenital syphilis is the most devastating complication of syphilis in pregnancy. The manifestation of congenital syphilis depends on many factors; gestational age, stage of maternal syphilis, maternal treatment and immunological response of the fetus.^5^ Pregnancies complicated by syphilis may result in intra-uterine growth restriction, non-immune hydrops fetalis, stillbirth, preterm delivery and spontaneous abortion^4^. In our cases, two different fetus outcomes were seen. In Case 1 no obvious clinical features of congenital syphilis were seen while in Case 2, the patient had a stillbirth.

Syphilis in pregnancy is diagnosed in a similar way to the non-pregnant population. Serological tests remain the mainstay for the diagnosis whereby the tests can be divided into two main categories namely non-treponemal tests (i.e. RPR, VDRL) and specific treponemal antibody tests. In our laboratory, we use RPR as our screening laboratory test for syphilis, which is further confirmed by treponemal-based test; syphilis IgM and IgG. Antenatal laboratory test for syphilis plays an important role for the diagnosis, as it is clearly shown that the timing of antenatal care interventions makes a significant difference in the risk of having an adverse outcome due to syphilis.^6^ High RPR titer at diagnosis is associated with increased risk of vertical transmission.^7^ It is also evident that those who are persistently negative in non-treponemal test will not transmit syphilis vertically.^8^ Early detection of syphilis will prompt early treatment to the patient thus reducing the risk of congenital syphilis as in Case 1. In Case 2, the diagnosis of syphilis was made after the complications occurred.

Multidiscipline approach involving obstetrician and pediatrician are required for management of syphilis in pregnancy. Penicillin is the mainstay of treatment for syphilis and given appropriately for the woman’s stage of syphilis.^3^^,^^5^^,^^7^ Parenteral rather than oral treatment has been the route of choice as the therapy is supervised with guaranteed bioavailability.^5^ The patient in Case 1 received appropriate treatment according to the stage of syphilis. Unfortunately, patient in Case 2 refused treatment at the time of writing. Besides penicillin, other antibiotics can be used to treat syphilis such as doxycycline and tetracycline, however are contraindicated during pregnancy.^3^ Following the diagnosis of syphilis, pregnant women should have monthly clinical and serological examination until delivery and thereafter follow up as in non-pregnant patients.^3^ Management of the sexual partner is also an important aspect for syphilis treatment and prevention.^3^^,^^7^ However, in both of our cases, the husbands refused for syphilis screening. 

In conclusion, syphilis is easily diagnosed with non-expensive tests available. Syphilis can be treated with an effective drug, penicillin, which is also used for the prevention of congenital syphilis. Yet, it remains a significant public health problem globally, including Malaysia. Effort to increase the awareness on the extent and gravity of syphilis in pregnancy is required at all levels of the health services, supported by high-level commitment.

## Authors Contribution:

AAW & UKI retrieved patient’s data and formulate the manuscript.

MM advised on the laboratory tests conducted on the patients.

EMMM & MMR help to review and editing the manuscript.

AAW takes the responsibility and is accountable for all aspects of the work to ensure the accuracy and integrity of the work are appropriately investigated and resolved. 
